# Toxicities of different first-line chemotherapy regimens in the treatment of advanced ovarian cancer: A network meta-analysis

**DOI:** 10.1097/MD.0000000000005797

**Published:** 2017-01-13

**Authors:** Chang-Ping Qu, Gui-Xia Sun, Shao-Qin Yang, Jun Tian, Jin-Ge Si, Yi-Feng Wang

**Affiliations:** a Department of Gynecology & Obstetrics, Huaihe Hospital of Henan University, Kaifeng; b Department of Gynecology and Obstetrics, Southern Medical University, Guangzhou, P.R. China.

**Keywords:** advanced ovarian cancer, Bayesian network model, chemotherapy, pharmacotherapy, randomized controlled trials, toxicity

## Abstract

**Background:**

Ovarian cancer (OC) is the 5th leading cause of cancer-related deaths around the world, and several chemotherapy regimens have been applied in the treatment of OC. We aim to compare toxicities of different chemotherapy regimens in the treatment of advanced ovarian cancer (AOC) using network meta-analysis.

**Methods:**

Literature research in Cochrane Library, PubMed, and EMBASE was performed up to November 2015. Eligible randomized controlled trials (RCTs) of different chemotherapy regimens were included. Network meta-analysis combined direct and indirect evidence to assess pooled odds ratios (ORs) and draw the surface under the cumulative ranking (SUCRA) curves.

**Results:**

Thirteen eligible RCTs were included in this network meta-analysis, including 8 chemotherapy regimens (paclitaxel + carboplatin [PC], pegylated liposomal doxorubicin [PLD] + carboplatin, carboplatin, gemcitabine + carboplatin, paclitaxel, PC + epirubicin, PC + topotecan, docetaxel + carboplatin). Gemcitabine + carboplatin regimen exerted higher incidence of anemia when compared with carboplatin and paclitaxel regimens. The incidence of febrile neutropenia of gemcitabine + carboplatin regimen was higher than that of PC, PLD + carboplatin, carboplatin, and PC + topotecan regimens. Topotecan PC + epirubicin regimen had a higher toxicity, comparing with PC, PLD + carboplatin, and PC + topotecan regimens. As for thrombocytopenia, gemcitabine + carboplatin chemotherapy regimen produced an obviously higher toxicity than PC and carboplatin. As for nausea, PLD + carboplatin chemotherapy regimen had a significantly higher toxicity than that of carboplatin chemotherapy regimen. Moreover, when compared with PC and carboplatin chemotherapy regimens, the toxicity of PC + epirubicin was greatly higher to patients with AOC.

**Conclusion:**

The nonhematologic toxicity of PLD + carboplatin regimen was higher than other regimens, which was clinically significant for the treatment of AOC.

## Abstract 2

Supplemental Digital Content is available in the text

## 1 Introduction

Ovarian cancer (OC) ranks top 2 in lethal gynecologic malignancy and is the 5th leading cause of cancer-related deaths around the world.^[1]^ However, the origin and pathogenesis of OC are still poorly understood. Studies have shown that OC is not a single disease, and various factors including diet, air stratification, industrial pollution, pathogen, and unhealthy living habits such as smoking are involved.^[2,3]^ Moreover, the prognosis for this disease is rather poor with a 5-year survival rate of only 30% to 40%, which is mainly caused by the lack of effective treatments.^[4]^ Although scanning is performed to detect and follow up the status of OC, there are still no effective means for its early detection. Furthermore, the unclear pathogenesis makes OC one of lethal diseases till now.

Currently, the treatment for OC mainly relies on chemotherapy. There are various chemotherapy agents, mainly including agents causing direct injury to cancer cells via cytotoxicity and to inhibit the growth of cancer cells, such as Paclitaxel, Pegylated Liposomal Doxorubicin (PLD),^[5,6]^ as well as agents inhibiting key molecule in a related signaling pathway to suppress proliferation and differentiation of cancer cells, such as Gemcitabine and Topotecan.^[7,8]^ In terms of other inhibition, Epirubicin, a cytotoxic chemotherapy agent, suppresses cancer cell proliferation by inhibition of DNA and RNA synthesis.^[9]^ Docetaxel makes cancer cells more likely to be identified and destroyed by T cells through changing cancer cell phenotype.^[10]^ Besides, it is fairly common to combine different drugs in order to enhance the inhibition of cancer cells as well as to reduce the side effects. As a frequently used match agent, Carboplatin is mainly used to weaken toxicity of conventional chemotherapy agents.^[11]^ Combination of Carboplatin with Paclitaxel, Docetaxel, and Gemcitabine are widely used in the treatment of OC as combined chemotherapies.^[2,12]^ However, there still needs a comprehensive study on comparing treatments of these chemotherapy agents. Pair-wise meta-analysis is frequently used to analyze data from randomized controlled trials (RCTs) in current clinical researches, which is poor at comparison of multiple factors and is often limited in the results.^[13]^ However, network meta-analysis could reintegrate and analyze interested intervene experiments and perform the comprehensive analysis of more than 1 intervention so as to obtain valuable and integrated results.^[14]^ Therefore, we performed current network meta-analysis to compare and assess toxicities of 8 chemotherapy regimens to human body in the treatment of advanced OC (AOC).

## 2 Materials and methods

### 2.1 Ethics statement

This study is a Network Meta-analysis and ethics statement is not applicable.

### 2.2 Literature search

Cochrane Library, PubMed, and EMBASE were searched by computer from the inception of each database to November 2015. The search was conducted using keywords combined free words including OC, pharmacotherapy, chemotherapy, Paclitaxel, Carboplatin, Topotecan, and so on.

### 2.3 Inclusion and exclusion criteria

The inclusion criteria included: (1) study design: RCT; (2) interventions: PC, PLD + Carboplatin, Carboplatin, Gemcitabine + Carboplatin, Paclitaxel, PC + Epirubicin, PC + Topotecan and Docetaxel + Carboplatin; (3) study subjects: patients with AOC aged 19 to 84 years; (4) outcomes: anemia, febrile neutropenia, thrombocytopenia, nausea, vomiting, fatigue, and diarrhea. The exclusion criteria included: (1) studies without sufficient data (nonmatch researches); (2) non-RCTs; (3) duplicated publications; (4) conference reports, system assessments or abstracts; (5) studies unrelated to the treatment of AOC; (6) non-English literatures; (7) nonhuman researches; (8) nonpharmacotherapy.

### 2.4 Data extraction and quality evaluation

Two reviewers independently extracted information from enrolled studies using uniform data collection sheets. In addition, other reviewers were consulted if these 2 reviews cannot reach an agreement. RCTs were assessed by more than 2 reviewers using Cochrane Collaboration's tool for assessing risk of bias, including sequence generation, allocation concealment, blinding, incomplete outcome data, selective outcome reporting, and other sources of bias.^[15]^ The assessment includes assigning a judgment of “yes,” “no,” or “unclear” for each domain to designate a low, high, or unclear risk of bias, respectively. The study was classified as a low risk of bias with less than 1 domain as low risk, whereas the study was assessed as high risk of bias if more than 4 fields were designed as high or unclear risk. In the rest situation, the study was deemed as the moderate risk of bias.^[16]^ Quality assessment and publication bias were carried out by Review Manager 5 (RevMan 5.2.3, Cochrane Collaboration, Oxford, UK).

### 2.5 Statistical analysis

First, directly compared different treatment arms were conducted using a pairwise meta-analysis. The data were presented with odd ratios (ORs) with 95% confidence intervals (CIs). Heterogeneity test was assessed using the chi-square test and *I*-square test.^[17]^ Second, R3.2.1 was conducted to draw net-like relation graph, in which each node refers to various intervention, node size refers to sample size, and line thickness between nodes refers to the number of enrolled studies. Then, Bayesian network meta-analyses were conducted. Each analysis was based on noninformative priors for effect sizes and precision. We also checked and confirmed the convergence and lack of auto correlation after 4 chains and a 20,000-simulation burn-in phase. Subsequently, direct probability statements were derived from an additional 50,000-simulation phase.^[18]^ The node-splitting method was carried out to assess the consistency between direct evidences and indirect evidences, and consistency or inconsistency model was selected on the basis of the results.^[17]^ In order to assist in the interpretation of weighted mean differences (WMDs), the probability of each intervention was calculated which was the most effective or safest treatment method summarized as surface under the cumulative ranking curve (SUCRA). The larger the SUCRA value suggested for a better rank of the intervention.^[19,20]^ All computations were done using R (V.3.2.1) package gemtc (V.0.6), supplemented with the Markov Chain Monte Carlo engine Open BUGS (V.3.4.0).

## 3 Results

### 3.1 Baseline characteristics of included study

**Figure 1:**
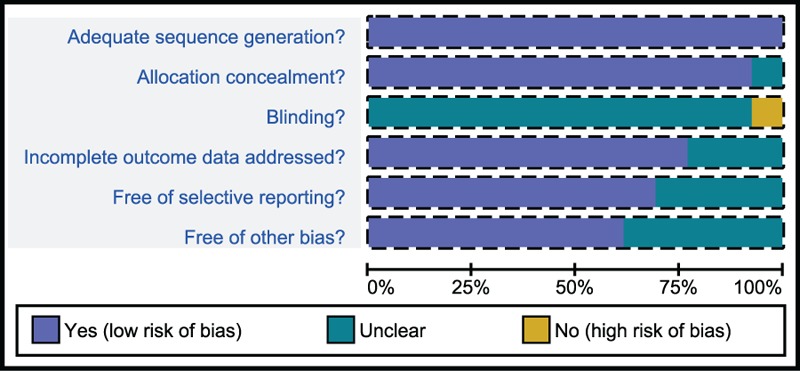
Cochrane risk of bias assessment map of included studies.

Through electronic databases, 2583 studies were identified. After the initial screening, we excluded 29 studies for duplication, 648 for letters or summaries, 180 for nonhuman studies, 144 for non-English studies. The remaining 1582 studies were assessed according to their full texts, and we further excluded 650 noncohort studies, 556 studies irrelevant to AOC, 360 studies irrelevant to chemotherapies, and 3 studies with no data or insufficient data. Eventually, 13 eligible RCTs,^[21–33]^ published between 2004 and 2015, were included for this network meta-analysis (Supplementary Figure 1). Totally, these 13 RCTs included 7841 patients with AOC, and the vast majority of patients received paclitaxel + carboplatin chemotherapy regimen. In 13 enrolled studies, 12 studies were from Europeans and 1 study was from Asians. Moreover, all 13 enrolled studies were 2-arm trials. The baseline characteristics of included studies were displayed in Table 1, and Cochrane risk of bias assessment was shown in Fig. 1.

**Table 1:**
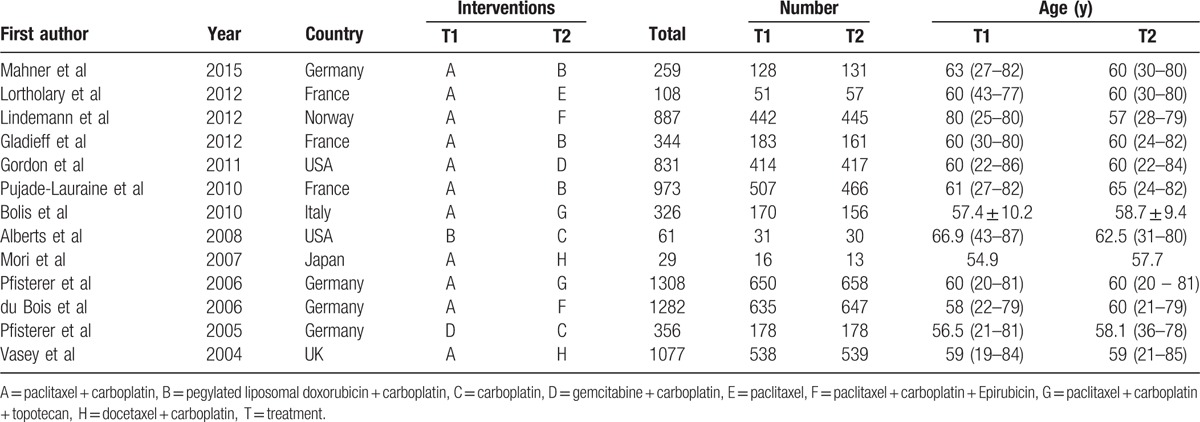
The baseline characteristics for included studies.

### 3.2 Pairwise meta-analysis for toxicities of 8 chemotherapy regimens in the treatment of AOC

We conducted direct paired comparisons for toxicities of 8 chemotherapy regimens in the treatment of AOC, and the results suggested that in terms of anemia, thrombocytopenia, and nausea, the toxicity of PC chemotherapy regimen was significantly lower when compared with PLD + carboplatin chemotherapy regimen (OR = 0.65, 95%CI = 0.43 – 0.98; OR = 0.49, 95%CI = 0.25 – 0.96; OR = 0.59, 95%CI = 0.45 – 0.77, respectively). With respect to anemia, febrile neutropenia, and thrombocytopenia, the toxicity of PC chemotherapy regimen patients with AOC was greatly lower than that of gemcitabine + carboplatin chemotherapy regimen (OR = 0.22, 95%CI = 0.14 – 0.33; OR = 0.08, 95%CI = 0.04 – 0.15; OR = 0.16, 95%CI = 0.11 – 0.23, respectively). While concerning anemia and thrombocytopenia, the gemcitabine + carboplatin chemotherapy regimen had a relatively higher toxicity to patients with AOC when compared with the carboplatin chemotherapy regimen (OR = 4.45, 95%CI = 2.35 – 8.41; OR = 6.26, 95%CI = 3.35 – 11.72, respectively). As for febrile neutropenia and nausea, comparing with the PC + epirubicin chemotherapy regimen, the toxicity of PC chemotherapy to patients with AOC was obviously lower (OR = 0.18, 95%CI = 0.12 – 0.26; OR = 0.40, 95%CI = 0.27 – 0.59, respectively). In terms of anemia, the toxicity of PC chemotherapy regimen to patients with AOC was remarkably higher than that of Paclitaxel chemotherapy regimen (OR = 4.31, 95%CI = 1.11 – 16.67). Meanwhile, with respect to febrile neutropenia, the PC chemotherapy regimen had a significantly higher toxicity to patients with AOC when compared with PC + topotecan chemotherapy regimen as well (OR = 3.87, 95%CI = 1.50 – 9.98) (Table 2). Furthermore, in reference to diarrhea, the toxicity of PC chemotherapy regimen to patients with AOC was greatly lower than that of docetaxel + carboplatin chemotherapy regimen (OR = 0.49, 95%CI = 0.26 – 0.90), and as for vomiting, comparing with PLD + carboplatin chemotherapy regimen, PC chemotherapy regimen had a relatively lower toxicity to patients with AOC (OR = 0.63, 95%CI = 0.46 – 0.88) (Supplementary Table 1).

**Table 2:**
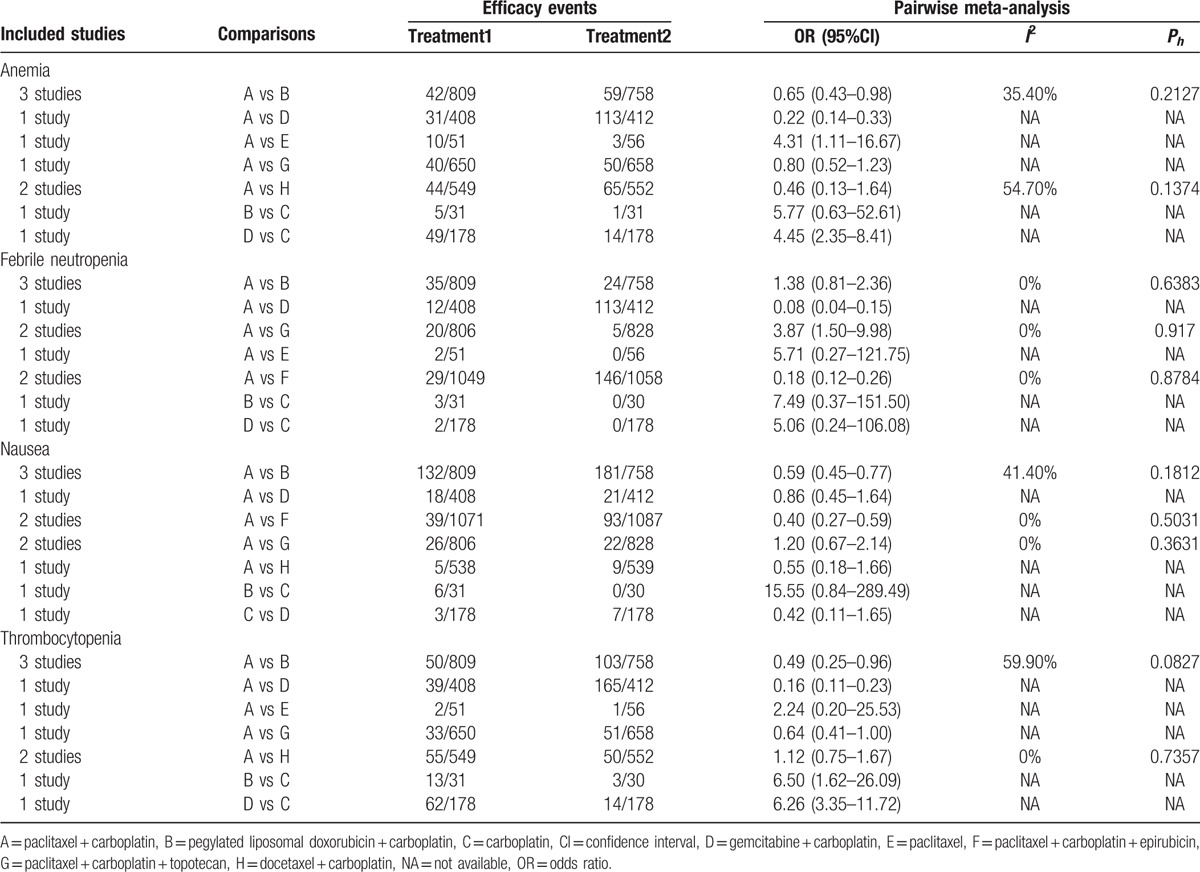
Estimated OR and 95%CI from pairwise meta-analysis in terms of anemia, febrile neutropenia, thrombocytopenia, and nausea.

### 3.3 Evidence network of 8 chemotherapy regimens in the treatment of AOC

**Figure 2:**
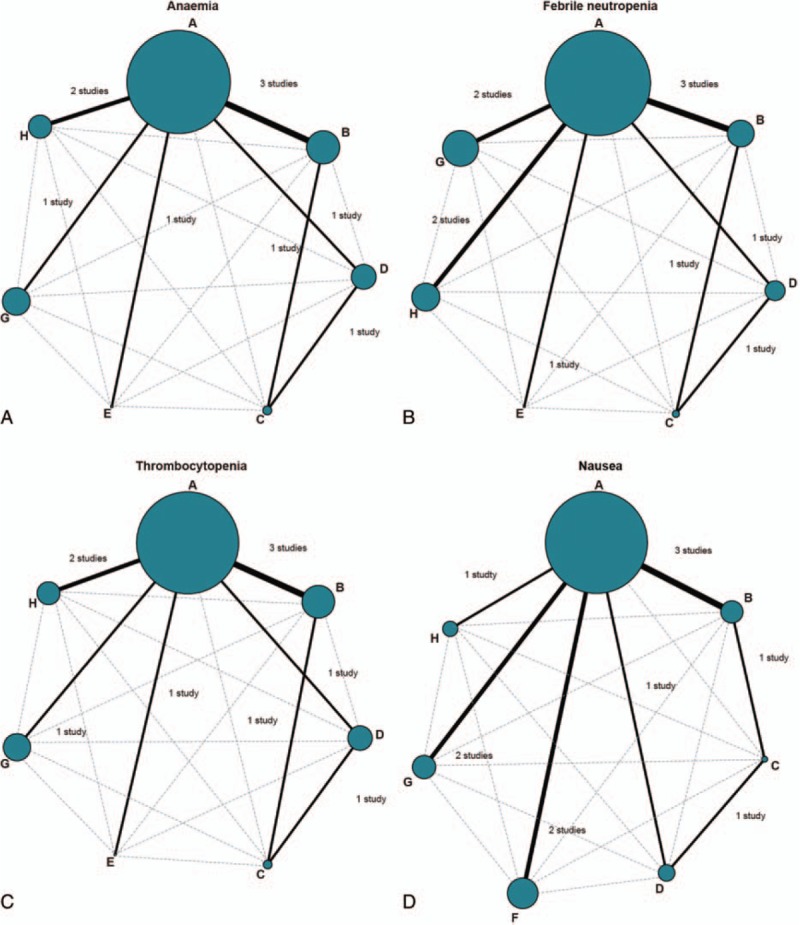
Evidence graph of anemia, febrile neutropenia, thrombocytopenia, and nausea (A = paclitaxel + carboplatin, B = pegylated liposomal doxorubicin + carboplatin, C = carboplatin, D = gemcitabine + carboplatin, E = paclitaxel, F = paclitaxel + carboplatin + epirubicin, G = paclitaxel + carboplatin + topotecan, H = docetaxel + carboplatin).

This study consisted of 8 chemotherapy regimens, that is, PC, PLD plus carboplatin, carboplatin, gemcitabine plus carboplatin, paclitaxel, PC plus epirubicin, PC plus topotecan and docetaxel plus carboplatin. With respect to anemia, febrile neutropenia, thrombocytopenia, nausea, vomiting, fatigue, and diarrhea, the largest number of patients with AOC received paclitaxel + carboplatin chemotherapy regimen. Additionally, the direct comparison between PC chemotherapy regimen and PLD + carboplatin chemotherapy regimen was relatively more (Fig. 2 and Supplementary Figure 2).

### 3.4 Inconsistency test of anemia, febrile neutropenia, thrombocytopenia, nausea, vomiting, fatigue, and diarrhea among all included studies

The node-splitting method was carried out for the inconsistency test of anemia, febrile neutropenia, thrombocytopenia, nausea, vomiting, fatigue, and diarrhea. The results illustrated that direct and indirect evidences of all outcome indicators were consistent. The consistency model was adopted (all *P* > 0.05) (Table 3).

**Table 3:**
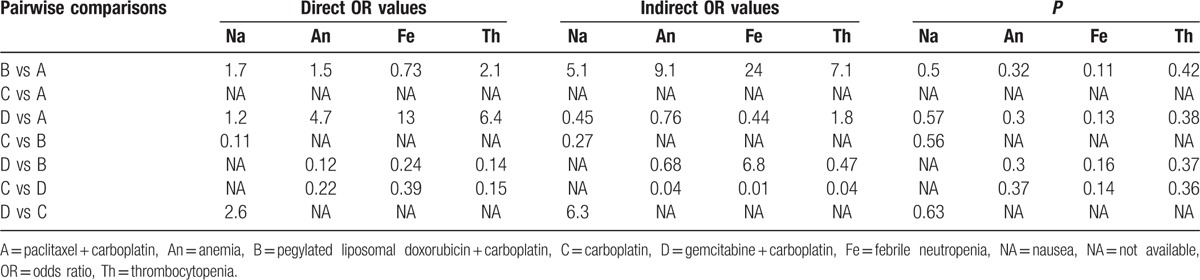
OR values and *P* values of direct and indirect pairwise comparisons of 4 treatment modalities under 4 endpoint outcomes.

### 3.5 Pooled results of network meta-analysis

**Figure 3:**
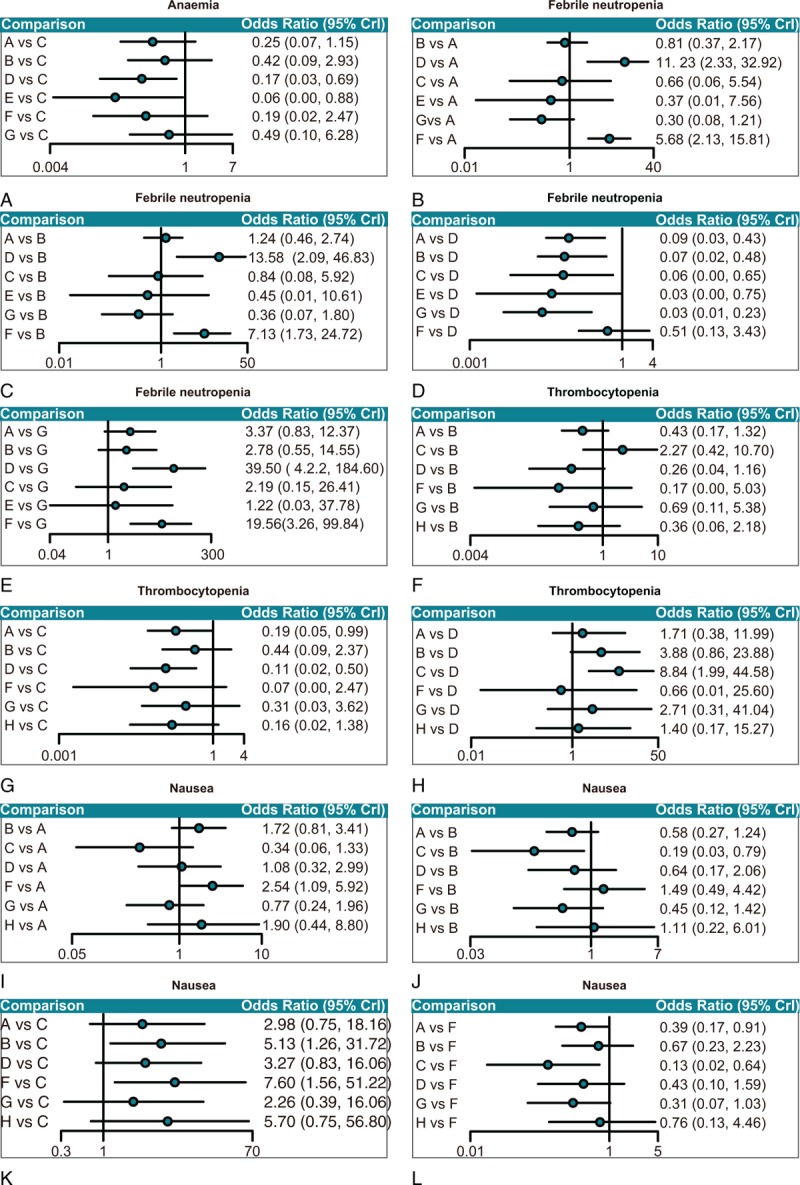
Forest map of correlation of anemia, febrile neutropenia, thrombocytopenia, and nausea (A = paclitaxel + carboplatin, B = pegylated liposomal doxorubicin + carboplatin, C = carboplatin, D = gemcitabine + carboplatin, E = paclitaxel, F = paclitaxel + carboplatin + epirubicin, G = paclitaxel + carboplatin + topotecan, H = docetaxel + carboplatin).

Network meta-analysis results revealed that with respect to hematologic toxicity, comparing with carboplatin and paclitaxel chemotherapy regimens, the toxicity of gemcitabine + carboplatin chemotherapy regimen was significantly higher to patients with AOC in terms of anemia (OR = 5.85, 95%CI = 1.45 – 34.70; OR = 18.09, 95%CI = 1.14 – 263.56, respectively). As for febrile neutropenia, the gemcitabine + carboplatin chemotherapy regimen had a greatly higher toxicity to patients with AOC when compared with PC, PLD + carboplatin, carboplatin, paclitaxel, and PC + topotecan chemotherapy regimens (OR = 11.23, 95%CI = 2.33 – 32.92; OR = 13.58, 95%CI = 2.09 – 46.83; OR = 15.96, 95%CI = 1.54 – 203.57; OR = 30.51, 95%CI = 1.33 – 1158.54; OR = 39.50, 95%CI = 4.42 – 184.60, respectively). Moreover, the toxicity of PC + epirubicin chemotherapy regimen was remarkably higher to patients with AOC than that of PC, PLD + carboplatin, and PC + topotecan chemotherapy regimens (OR = 5.68, 95%CI = 2.13–15.81; OR = 7.13, 95%CI = 1.73–24.72; OR = 19.56, 95%CI = 3.26–99.84, respectively). As for thrombocytopenia, gemcitabine + carboplatin chemotherapy regimen exerted obviously higher toxic effects on patients with AOC when compared with PC and carboplatin chemotherapy regimens (OR = 5.29, 95%CI = 1.00 – 20.30; OR = 8.84, 95%CI = 1.99 – 44.58, respectively) (Supplementary Table 2 and Fig. 3). With respect to nonhematologic toxicity, when concerning nausea, the toxicity of PLD + carboplatin chemotherapy regimen was significantly higher to patients with advanced ovarian cancer than that of the carboplatin chemotherapy regimen (OR = 5.13, 95%CI = 1.26 – 31.72). Moreover, comparing with PC and carboplatin chemotherapy regimens, the PC + epirubicin chemotherapy regimen exerted relatively higher toxic effects on patients with AOC (OR = 2.54, 95%CI = 1.09 – 5.72; OR = 7.60, 95%CI = 1.56 – 51.22, respectively) (Supplementary Table 3 and Fig. 3). However, in terms of vomiting, fatigue, and diarrhea, there were no significant differences among toxicities of these 8 chemotherapy regimens to AOC (Supplementary Table 3).

### 3.6 SUCRA curves of the toxicity of 8 chemotherapy regimens in the treatment of AOC

As shown in Table 4, in SUCRA values of 8 chemotherapy regimens, the lowest SUCRA value of the incidence of fatigue (38.3%), anemia (22.0%), febrile neutropenia (17.0%), and thrombocytopenia (19.6%) was gemcitabine + carboplatin chemotherapy regimen. Besides, the PC + epirubicin chemotherapy regimen achieved the lowest SUCRA value of the incidence of nausea (23.1%). However, the PLD + carboplatin regimen showed lower SUCRA value of vomiting (30.0%) and the docetaxel + carboplatin regimen had lower SUCRA value of diarrhea (29.2%) than other regimens. Generally, the incidence of hematologic toxicity of gemcitabine + carboplatin regimen was highest for AOC patients, and PC + epirubicin, PLD + carboplatin, and docetaxel + carboplatin regimens had higher incidence of nonhematologic toxicity for AOC patients.

**Table 4:**
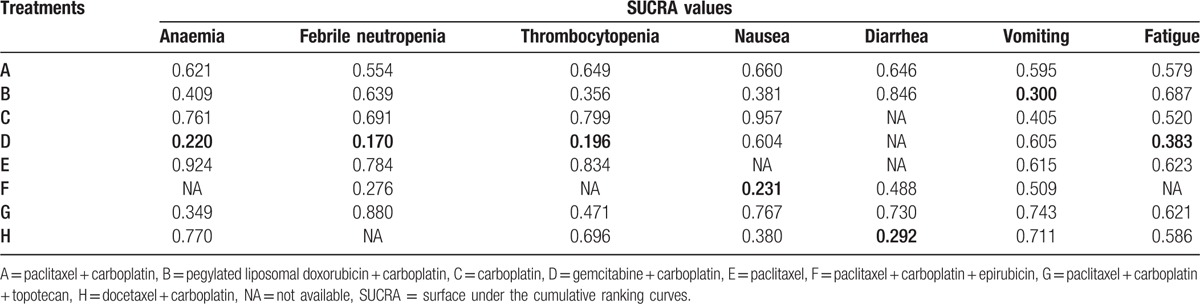
SUCRA values of 11 treatment modalities under 6 endpoint outcomes.

## 4 Discussion

This study mainly aimed to analyze 8 chemotherapy regimens in the treatment of AOC. The direct pairwise meta-analysis and network meta-analysis results demonstrated that the incidence of nonhematologic toxicity of AOC patients treated with the PLD + carboplatin chemotherapy regimen was higher than other chemotherapy regimens. The main toxicities that may occur in AOC patients treated with PLD chemotherapy regimen are nausea, palmar-plantar erythema or hand-foot syndrome, stomatitis, and myelosuppression.^[34]^ Some recent studies showed that the PLD + carboplatin chemotherapy regimen had significantly higher incidence of anemia and thrombocytopenia, and AOC patients receiving PLD + carboplatin chemotherapy regimen had higher incidence of experiencing dose delays than those in the standard treatment arm and may had discontinued treatment because of toxicity or refusal.^[35]^ Accordingly, PLD + carboplatin chemotherapy regimen showed more nonhematologic toxicity for AOC patients.

Direct pairwise meta-analysis results also revealed that the gemcitabine + carboplatin chemotherapy regimen was the most toxic regimen in hematologic for AOC patients among 8 chemotherapy regimens. Specifically, anemia, febrile neutropenia, and thrombocytopenia had larger OR and 95%CI. Furthermore, network meta-analysis results also confirmed that gemcitabine + carboplatin chemotherapy regimen was obviously correlated with these toxic effects. Currently, several researches reported that the toxicity of carboplatin-based treatment was relatively low and unapparent.^[36,37]^ Therefore, it was speculated that gemcitabine was more likely to cause the toxic effects of the carboplatin + gemcitabine chemotherapy regimen. Besides, current researches still cannot reach agreement on the toxic effects of gemcitabine. On the one hand, some researchers reported that the toxicity of gemcitabine was comparatively mild, and clinical data displayed that only 5% patients in early phase I and phase II had neutropenia when using gemcitabine-based treatment.^[38,39]^ On the other hand, there was a study revealing that the occurrence rate of toxic reactions of gemcitabine was relatively high in advanced cancer patients, namely, neutropenia in toxic effects of gemcitabine was 18%, thrombocytopenia of 16%, and anemia of 10%.^[40]^ Hence, gemcitabine has significant toxic effects on advanced cancer patients, which is consistent with our analysis results. Gemcitabine mainly functions by inhibiting poly-ADP-ribose polymerase (PARP) to interfere with the proliferation and differentiation of cancer cells.^[7]^ Meanwhile, PARP also plays an important role in regulating the proliferation and differentiation of normal cells.^[41]^ Consequently, gemcitabine has less toxic effects on patients in early stage since their overall physiological status is normal. However, for patients in the advanced stage, gemcitabine will indirectly inhibit the cell repair and generates more toxic effects.

Pairwise meta-analysis and network meta-analysis results showed that the toxicity of PC chemotherapy regimen was lower than that of the other 7 chemotherapy regimens. PC is 1 of common first-line chemotherapy regimens in the treatment of OC. As an anticancer drug, paclitaxel binds specifically in reversible manner to N-terminal 31 amino acids to the beta-tubulin subunit in the microtubules, which later inhibits microtubule formation.^[5]^ For this reason, paclitaxel could restrict the effect that cancer cells strengthen their proliferation and metastasis by hyperplasia of capillaries.^[42]^ With a view to the specific treatment target of paclitaxel, it is reasonable that paclitaxel produces lower toxicity to human body. Recently, clinical research results certified that the toxicity of PC + carboplatin chemotherapy regimen was lower when compared with PC + topotecan, docetaxel + carboplatin, and paclitaxel chemotherapy regimens.^[2,12,43]^ In the meantime, network meta-analysis also proved that the toxic effects of PC + epirubicin chemotherapy regimen was low, second only to gemcitabine + carboplatin chemotherapy regimen, which was caused by the mechanism of epirubicin action, that is, epirubicin inserts into DNA double-strand to block synthesis of DNA and RNA.^[9]^ In consequence, epirubicin is able to inhibit cancer cell proliferation, but it could also cause great injury to normal cells.

Methodologically, the Bayesian network model was conducted for the inconsistency test of direct and indirect evidences via the node splitting method. By this method, we could eliminate the potential errors in network meta-analysis and further conduct comparison under various interventions, which makes experimental data more accurate.^[44]^ Nevertheless, several limitations deserve our attentions. First, the number of included literatures was relatively small, which will make this study far less diversified, and there was no cross-research comparison, which constrains the university of conclusion. Second, some differences in sample size of 8 interventions, which may have a certain impact on the accuracy, besides, the majority comparison were between paclitaxel + carboplatin and PLD + carboplatin regimens, which easily lead to the conclusion about that PLD + carboplatin had the highest incidence of hematologic toxicity for AOC patients.^[45,46]^ However, due to the large quantity of patients enrolled in this study and the consistency with current research progress, the conclusion is valuable and significant.

In conclusion, this study clearly demonstrated that the PLD + carboplatin chemotherapy regimen exerts the highest toxic effects in hematologic on patients with AOC, and it is clinically significant for the future clinical medication and therapy development.

## Supplementary Material

Supplemental Digital Content

## Supplementary Material

Supplemental Digital Content

## Supplementary Material

Supplemental Digital Content
