# Supplementary material for: Toxicities of different first-line chemotherapy regimens in the treatment of advanced ovarian cancer: A network meta-analysis
Source: Medicine (Baltimore). 2017 Jan 13;96(2):e5797. doi: 10.1097/MD.0000000000005797 (PMC5266167; doi:10.1097/MD.0000000000005797)
Supplement: Supplemental Digital Content [file medi-96-e5797-s001.doc]

**Supplementary FIG. 1.** Flow chart of literature screening

**Supplementary FIG. 2.** Evidence graph of vomiting, fatigue and diarrhea (A = Paclitaxel + Carboplatin; B = pegylated liposomal doxorubicin + Carboplatin; C = Carboplatin; D = Gemcitabine + Carboplatin; E = Paclitaxel; F = Paclitaxel + Carboplatin + Epirubicin; G = Paclitaxel + Carboplatin + Topotecan; H = Docetaxel + Carboplatin)

**
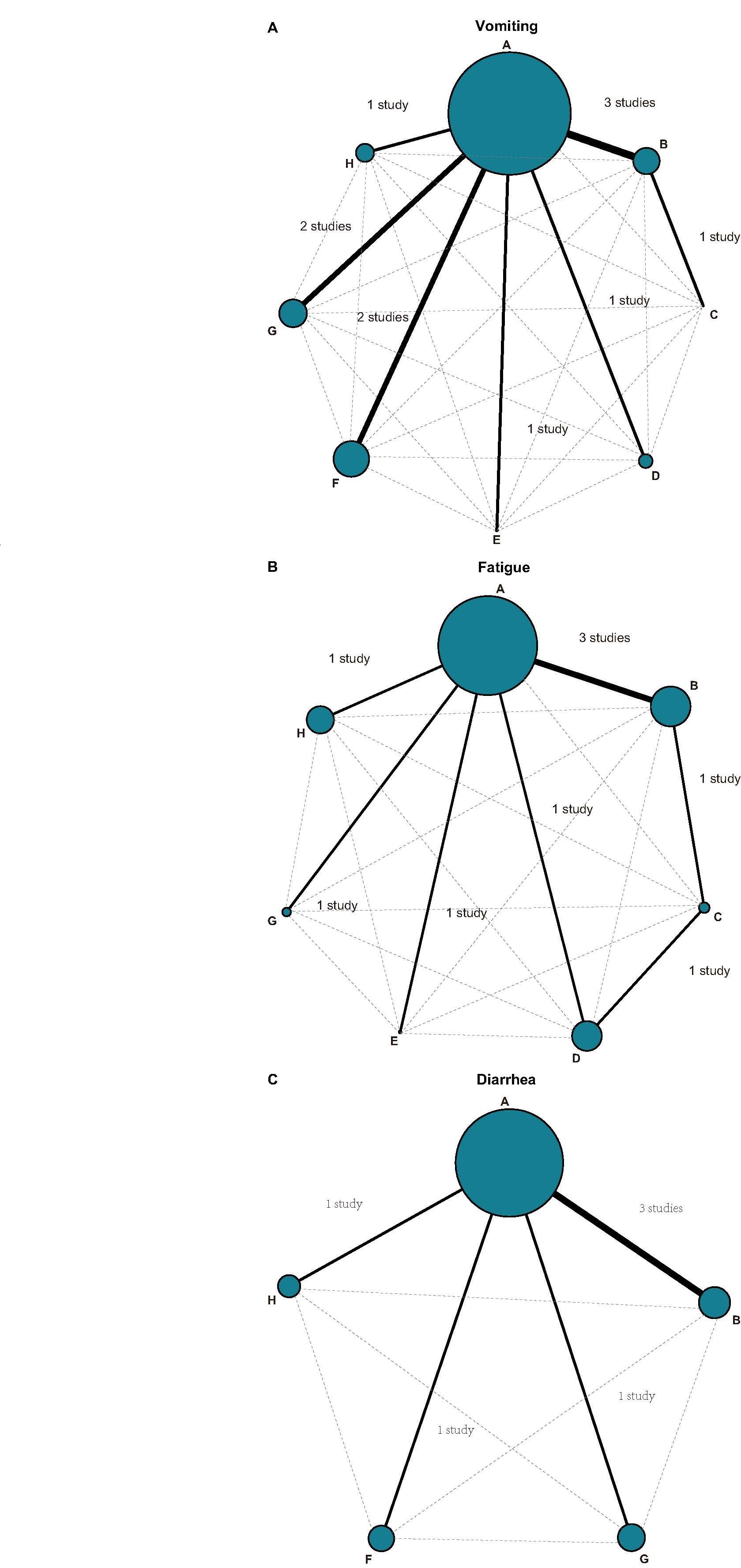
**

**Appendix table 1.** Estimated OR and 95%CI from pairwise meta-analysis in terms of vomiting, fatigue and diarrhea.

| **Included studies** | **Comparisons** | **Efficacy events** | |  | **Pairwise meta-analysis** | | |
| --- | --- | --- | --- | --- | --- | --- | --- |
|  |  | **Treatment1** | **Treatment2** |  | **OR (95%CI)** | ***I*^2^** | ***P_h_*** |
| **Diarrhea** | | | | | | | |
| 3 studies | A vs. B | 53/809 | 36/758 |  | 1.41 (0.91-2.18) | 0.0% | 0.527 |
| 1 study | A vs. G | 17/650 | 15/658 |  | 1.15 (0.57-2.23) | NA | NA |
| 1 study | A vs. F | 17/611 | 23/622 |  | 0.75 (0.39-1.41) | NA | NA |
| 1 study | A vs. H | 16/538 | 32/539 |  | **0.49 (0.26-0.90)** | NA | NA |
| **Vomiting** | | | | | | | |
| 3 studies | A vs. B | 92/809 | 126/758 |  | **0.63 (0.46-0.88)** | 39.5% | 0.192 |
| 1 study | A vs. D | 17/408 | 16/412 |  | 1.08 (0.54-2.16) | NA | NA |
| 1 study | A vs. E | 10/51 | 10/56 |  | 1.12 (0.42-2.97) | NA | NA |
| 2 studies | A vs. F | 77/1047 | 87/1064 |  | 0.78 (0.27-2.23) | 89.0% | 0.003 |
| 2 studies | A vs. G | 18/806 | 14/828 |  | 1.27 (0.62-2.61) | 28.3% | 0.238 |
| 1 study | A vs. H | 70/538 | 43/539 |  | 1.73 (1.16-2.57) | NA | NA |
| 1 study | B vs. C | 1/31 | 0/31 |  | 3.00 (0.12-76.58) | NA | NA |
| **Fatigue** | | | | | | | |
| 3 studies | A vs. B | 224/809 | 192/758 |  | 1.13(0.89-1.44) | 0.0% | 0.736 |
| 1 study | A vs. D | 15/408 | 23/412 |  | 0.65 (0.33-1.26) | NA | NA |
| 1 study | A vs. E | 31/51 | 33/56 |  | 1.08 (0.50-2.34) | NA | NA |
| 1 study | A vs. G | 1/156 | 0/170 |  | 3.29 (0.13-81.34) | NA | NA |
| 1 study | A vs. H | 43/538 | 43/539 |  | 1.00 (0.64-1.56) | NA | NA |
| 1 study | B vs. C | 3/31 | 2/30 |  | 1.50 (0.23-9.68) | NA | NA |
| 1 study | C vs. D | 3/178 | 4/178 |  | 1.34 (0.30-6.08) | NA | NA |

Notes: NA=not available; A= paclitaxel+carboplatin; B= pegylated liposomal doxorubicin+Carboplatin; C= Carboplatin; D= Gemcitabine+Carboplatin; E= Paclitaxel; F= paclitaxel+ carboplatin +Epirubicin; G= paclitaxel+carboplatin+Topotecan; H= Docetaxel+Carboplatin
